# AI-based computer vision as an adjunct tool for CVS documentation in laparoscopic cholecystectomy

**DOI:** 10.1007/s00464-026-12780-y

**Published:** 2026-04-16

**Authors:** Danit Dayan, Monica Ortenzi, Eran Nizri

**Affiliations:** 1 Division of General Surgery, Tel Aviv Medical Center, 6 Weizman St., 6423909 Tel Aviv, Israel; 2 Gray Faculty of Medical & Health Sciences, Tel Aviv University, Tel Aviv, Israel; 3 Department of Clinical and Emergency Surgery, Polytechnic University of Marche, Ancona, Italy

**Keywords:** Laparoscopic cholecystectomy, Artificial intelligence, Computer vision, Critical view of safety, Operative documentation

## Abstract

**Background:**

Despite widespread endorsement for Critical View of Safety (CVS) to prevent bile duct injury during laparoscopic cholecystectomy (LC), attainment remains low. Quality assurance is limited by inaccurate operative reports and absent visual documentation. This study evaluated a surgical artificial intelligence (AI) platform as an adjunct for CVS documentation, comparing its performance to narrative operative reports using expert video review as the reference-standard.

**Methods:**

This retrospective study included consecutive LC videos routinely captured and automatically analyzed by an implemented AI-based computer vision platform in a tertiary center (December 2022-August 2023). Three expert surgeons independently double-reviewed all videos, establishing ground-truth for CVS achievement by majority consensus. CVS classifications by the AI-platform and operative reports were compared against the ground-truth. Performance metrics were calculated and discriminatory performance was examined, due to class imbalance, using the area under the receiver operating characteristic curve (AUROC) and Matthews Correlation Coefficient (MCC). Analyses were stratified according to Parkland’s grading scale.

**Results:**

Among 279 LC videos, 83.5% involved high disease severity (Parkland 3–5). The overall intra-rater reliability was high (Cohen’s kappa > 0.8), and multi-rater reliability was moderate (Fleiss’ kappa = 0.58). Ground-truth indicated CVS achievement in 74.2% of cases, compared with 55.2% identified by the AI-platform and 92.5% documented in operative reports. Despite similar overall agreement (73.8% vs. 73.8%; p = 0.39), the AI-platform had higher reliability (κ 0.45 vs. 0.13; p < 0.001), reflecting differing sensitivity (69.6% vs. 99.0%) and specificity (86.1% vs. 10.6%). In the context of class imbalance, the AI-platform had significantly higher AUROC than operative reports (0.77 vs. 0.55; p < 0.001), with consistently superior discriminatory CVS documentation across disease severity. MCC was 0.49 for the AI-platform and 0.13 for operative reports.

**Conclusions:**

The AI-platform appears to have superior discriminatory performance and more reliable alignment with expert ground-truth than narrative documentation, supporting its use as an adjunct for CVS documentation.

## Introduction

Critical view of safety (CVS) is a key safety practice in laparoscopic cholecystectomy (LC) that aims to prevent bile duct injury [1], a rare but serious complication. This technique, originally described by Strasberg et al. [2], has been endorsed by surgical societies and incorporated into international guidelines for safe cholecystectomy [3, 4]. Nevertheless, CVS adoption rates remain suboptimal in clinical practice, reported as low as 10.8% [5–7]. Accurate documentation is essential for quality monitoring, surgeon feedback, and medico-legal accountability. However, narrative operative reports are often inadequate, limiting a reliable assessment of surgical safety practices [8–12].

This discrepancy highlights a fundamental challenge in validating safety practices when documentation is not directly verifiable. Artificial intelligence (AI)-based computer vision technologies offer a promising solution through operative video analysis to identify key safety steps, including CVS [13, 14]. In a previous real-world quality improvement initiative using an AI-based computer vision platform, we demonstrated an increase in CVS adoption from 39.2% to 69.2% within three months [15].

Building on this previous initiative, and given that operative reports remain the predominant form of documentation, the AI platform was compared with narrative operative reports, each evaluated against expert majority ground truth, to determine whether the platform provides more accurate and reliable CVS classification, as an adjunct to operative documentation, an aspect that has not been previously evaluated. We hypothesized that the platform would provide more reliable documentation, particularly in identifying cases where CVS was not achieved.

## Methods

This retrospective study included consecutive LCs performed at a large tertiary academic hospital between December 2022 and August 2023. All procedures were routinely video recorded and automatically analyzed using a surgical intelligence platform (Theator Inc., Palo Alto, California, USA), as previously described by Fried et al. [15]. This video cohort was not used for model training. The AI platform was implemented for routine use in our medical center and generated automated breakdown of the LC video into key-moments and annotations, including the CVS and disease severity scoring according to Parkland grading scale for cholecystitis [16, 17]. Based on the platform’s output, Parkland grades were stratified into low-severity (Parkland 1–2) and high-severity (Parkland 3–5) groups. In a separate study conducted by our group, the platform appeared to show good concordance for PGS grading, with F1 scores exceeding 0.9 across severity levels (unpublished data). The study was approved by the institutional review board (Approval Number TLV-0391–23) with a waiver of informed consent.

## AI-based CVS documentation

The AI platform automatically assessed CVS achievement in each video based on three mandatory criteria: (1) The hepatocystic triangle is cleared of fat and fibrous tissue, (2) The lower third of the gallbladder is separated from the liver to expose the cystic plate, and (3) Two and only two structures are seen entering the gallbladder. The platform provides a binary classification (yes/no) for each component, indicating complete CVS achievement when all three criteria are met. The algorithm applies predefined criteria across all cases.

## CVS documentation in narrative operative reports

Narrative operative reports were collected from the electronic medical records and reviewed to determine whether the CVS was achieved, not achieved, or not documented. Reports were evaluated strictly based on the written documentation, without inference or reinterpretation.

## Ground truth (expert majority consensus) for CVS achievement

Three expert surgeons independently reviewed all LC videos to determine CVS achievements. Each surgeon assessed all cases twice in separate sessions to calculate intra-rater reliability. Experts’ assessments were compared to determine inter-rater agreement, and majority consensus of at least two reviewers was used as the ground-truth reference-standard against which the AI platform and narrative operative reports were evaluated. Expert video review was selected as the most reproducible reference-standard despite inherent subjectivity.

## Statistical analysis and outcome measures

Statistical analyses were performed using IBM SPSS Statistics (version 29.0) and Jamovi (version 2.6.26.0) and were stratified according to Parkland’s grading scale when relevant.

Intra-rater and pairwise inter-rater reliability, as well as agreement between the AI platform and operative reports against the ground truth, were assessed using Cohen’s kappa with Chi-square testing for significance. Overall multi-rater reliability was evaluated using Fleiss’ kappa. A p-value < 0.05 was considered indicative of agreement and reliability beyond chance.

Performance metrics calculated for both the AI platform and narrative operative reports included overall agreement, reliability (κ), sensitivity, specificity, positive predictive value (PPV), and negative predictive value (NPV). For the AI platform, the F1 score was computed as 2 × (Precision × Recall) / (Precision + Recall), where Precision = PPV and Recall = Sensitivity. The McNemar test, a paired statistical test used to compare two binary classification methods by evaluating whether the proportion of discordant pairs differs significantly, was used to compare statistical differences between the AI platform and operative reports.

Discriminatory performance was examined, due to class imbalance, using receiver operating characteristic curve (ROC) and Matthews Correlation Coefficient (MCC). The area under the ROC (AUROC) was reported with 95% confidence intervals and p-values. Differences between methods were assessed using paired bootstrap resampling, which repeatedly samples the data to estimate confidence intervals, with 5,000 replicates used for formal statistical comparisons. MCC was reported as a descriptive measure of overall classification agreement, calculated as (TP·TN − FP·FN) / √[(TP + FP)(TP + FN)(TN + FP)(TN + FN)] without inferential testing. A criterion-level analysis was performed for each of the three CVS criteria, including Fleiss’ kappa, F1 score and AUROC.

## Results

The study cohort included a total of 279 LC videos that were routinely captured and analyzed by the AI platform. Most videos (*n* = 233, 83.5%) involved high disease severity (Parkland scores 3–5) with only a minority (*n* = 46, 16.5%) involving low disease severity (Parkland scores 1–2).

Table 1 summarizes intra-rater agreement for CVS achievement, stratified by Parkland. Agreements exceeded 96 and 80% for achieved CVS and not-achieved CVS, respectively. Cohen’s kappa demonstrated very high (> 0.8) intra-rater reliability for CVS repeated assessment across all experts, indicating consistent evaluations beyond chance (P < 0.001). Intra-rater reliability was consistently high in high-severity cases (κ 0.85–0.90) compared with low-severity cases (κ 0.60–1.00) with greater agreement on CVS not achieved (i.e., it was more readily discernible under clinical conditions of higher severity).

**Table 1:** Intra- rater agreement for CVS achievement in LC videos, stratified by Parkland

Rater	% Agreement on CVS Achievements		Cohen’s K	P-value
	Achieved	Not achieved		
Overall	N = 279			
Expert 1	96.9	85.2	.83	< 0.001
Expert 2	99.4	82.1	.84	< 0.001
Expert 3	99.5	90.2	.92	< 0.001
Parkland 1–2	N = 46 (16.5%)			
Expert 1	92.7	80	0.62	< 0.001
Expert 2	90.0	83.3	0.60	< 0.001
Expert 3	100	100	1	< 0.001
Parkland 3–5	N = 233 (83.5%)			
Expert 1	97.3	87.5	0.85	< 0.001
Expert 2	90.1	100	0.86	< 0.001
Expert 3	94.3	98.7	0.90	< 0.001

Table 2 summarizes the pairwise inter-rater and multi-rater agreements for CVS achievement, stratified by Parkland. Agreements were high (≥ 80%) across expert pairs, with higher agreement and reliability in high-severity cases (i.e., surgeons were more consistent in assessing CVS under clinical conditions of higher severity and specifically when CVS was not achieved). However, the Cohen’s kappa values were overall moderate (0.48) to high (0.63 and 0.64) beyond chance (P < 0.001), underscoring subjectivity when CVS was not achieved across Parkland grades, whereas CVS achieved was consistent.

**Table 2:** Inter- rater and multi-rater agreements for CVS achievement in LC videos, stratified by Parkland

A					
		Pairwise inter-rater agreement			
**Raters**	% Agreement on CVS achievements			Cohen’s K	*P*-value
	Achieved		Not Achieved		
**Overall**	*N* = 279				
Expert 1 vs. 2	98.9		58.0	0.64	< 0.001
Expert 1 vs. 3	93.8		48.8	0.48	< 0.001
Expert 2 vs. 3	87.7		76.2	0.63	< 0.001
**Parkland 1–2**	*N* = 46 (16.5%)				
Expert 1 vs. 2	100		83.3	0.9	< 0.001
Expert 1 vs. 3	94.7		37.5	0.38	< 0.001
Expert 2 vs. 3	94.7		50	0.50	< 0.001
**Parkland 3–5**	*N* = 233 (83.5%)				< 0.001
Expert 1 vs. 2	98.7		56.1	0.60	< 0.001
Expert 1 vs. 3	93.6		50.0	0.48	0.008
Expert 2 vs. 3	86.0		78.9	0.64	< 0.001

B					
		**Overall multi-rater agreement**			
	% Agreement on CVS achievements			Fleiss K (95% CI)	*P*-value
	3 of 3 raters		2 of 3 raters		
**Overall**	74.9		25.1	0.58 (0.51–0.65)	< 0.001
Parkland 1–2	84.8		15.2	0.57 (0.41–0.74)	< 0.001
Parkland 3–5	73.0		27.0	0.57 (0.50–0.65)	< 0.001

The overall multi-rater agreement consisted of 74.9% for unanimous ratings and 25.1% for two-expert agreement. Assessment using Fleiss’ kappa demonstrated moderate reliability (*k* = 0.58, 95% CI 0.51–0.65) across all three reviewers, supporting agreement beyond chance (P < 0.001). Despite similar reliability across disease severity, in high-severity cases the raters were more consistent, as indicated by a narrower 95% CI (0.57, 0.50–0.65 vs. 0.57, 0.41–0.74).

The ground-truth dataset included all 279 cases, of which 207 (74.2%) were classified as achieved and 72 (25.8%) as not-achieved CVS. Table 3 shows the distribution of correct and incorrect classifications for narrative operative reports and the AI platform relative to the ground truth. The AI platform classified 55.2% of cases as achieved CVS compared with 92.5% as documented in the operative reports. This pattern suggests systematic over documentation of achieved CVS in narrative reports.

**Table 3:** Frequency of CVS achievement according to (A) narrative operative reports and (B) the surgical AI platform, compared with expert ground truth in LC videos

A		**Operative reports**			
		Yes	No	Not documented	
Ground-truth	Yes	199	2	6	207
	No	59	7	6	72
		258	9	12	279
B Ground-truth		**AI Platform**			
		Yes	No	Not Documented	
	Yes	144	63	-	207
	No	10	62	-	72
		154	125	-	279

Ground-truth CVS achievement was defined by majority agreement (≥ 2 of 3 experts). The dataset had class imbalance with CVS Achieved predominance: ground-truth (74.2%), operative reports (92.5%), and AI platform (55.2%)

Performance metrics for the AI platform and operative reports are shown in Table 4. Compared with expert ground truth, overall agreements were equally high for both documentation methods (73.8 vs. 73.8%; *p* = 0.39). However, reliability differed significantly, with moderate Cohen’s kappa for the AI platform and low Cohen’s kappa for the operative reports (0.45 vs. 0.13; p < 0.001), consistent with the kappa paradox [18]. This paradox arises from prevalence bias, with achieved CVS predominating and further amplified by systematic overcalling in the operative reports. Under these conditions, even small disagreements in the minority class (i.e., not-achieved CVS) substantially suppress kappa, leading to an apparent reduction in reliability despite high overall agreement [18]. This effect applies for both the AI platform and the operative reports. Because overall agreement performs poorly in imbalanced datasets, the similar crude agreement masks the substantially different classification profiles of the two documentation methods. Operative reports demonstrated very high sensitivity (99.0%) but low specificity (10.6%), indicating a tendency to classify most cases as CVS achieved, whereas the AI platform showed a more balanced distribution of classifications, with sensitivity of 69.6% and specificity of 86.1%. Additional metrics were therefore examined to characterize discriminatory performance due to class imbalance.

**Table 4:** Performance of narrative operative reports and the surgical AI platform compared with ground- truth in documenting the CVS in LC

Performance metric	Operative reports	AI platform	*p*-value
Overall agreement, % Reliability, Cohen’s kappa	73.8 0.13	73.8 0.45	0.39 < 0.001
Sensitivity, %	99.0	69.6	
Specificity,%	10.6	86.1	
Positive predictive value,%	77.1	93.5	
Negative predictive value,%	77.8	49.6	
Algorithm F1 score	–	0.8	
AUROC *			
Overall	0.55 (95% CI 0.51–0.59)	0.77 (95% CI 0.72–0.82)	< 0.001
Parkland 1–2 (N = 46, 16.5%)	0.60 (95% CI 0.5–0.8)	0.87 (95% CI 0.80–0.94)	< 0.001
Parkland 3–5 (N = 233, 83.5%)	0.54 (95% CI 0.51–0.58)	0.76 (95% CI 0.70–0.82)	0.014
MCC *			
Overall	0.13	0.49	
Parkland 1–2 (N = 46, 16.5%)	0.43	0.53	
Parkland 3–5 (N = 233, 83.5%)	0.21	0.48	

^*^ AUROC and MCC were used to examine discriminatory performance due to class imbalance

Metrics used for this purpose included the AUROC and MCC. The AI platform demonstrated significantly (*p* < 0.001) superior discrimination (AUROC 0.77, 95% CI 0.72–0.82) than the operative reports (0.55, 95% CI 0.51–0.59). The AI-platform showed consistent high discriminatory performance across disease severity subgroups (0.76, 95% CI 0.70–0.82 in high severity and 0.87, 95% CI 0.80–0.94 in low severity) compared with lower discrimination as documented in the operative notes (0.54, 95% CI 0.51–0.58 in high severity and 0.60, 95% CI 0.5–0.8 in low-severity cases).

Accordingly, the overall MCC was 0.49 for the AI platform and 0.13 for operative reports and was consistent in the platform across disease severity classification (0.48 in high severity and 0.53 in low-severity cases) and inconsistent in the operative notes (0.21 in high severity and 0.43 in low-severity cases). The platform achieved F1 score of 0.8.

A criterion-level analysis showed that reliability and performance varied across the three CVS components. The platform matched majority rater consensus in 90.2% of cases for criterion (1), 89.2% for criterion (2), and 70.9% for criterion (3). Correspondingly, criterion (1) demonstrated *κ* = 0.29 with F1 = 0.95 and AUROC = 0.75; criterion (2) had *κ* = 0.42 with F1 = 0.94 and AUROC = 0.79; and criterion (3) had *κ* = 0.38 with F1 = 0.79 and AUROC = 0.78 (all *p* < 0.001). Low Fleiss’ kappa values mirror class imbalances (consistent with the kappa paradox), since most criteria were achieved. The relatively lower performance observed for criterion (3) may reflect the platform’s strict pre-dissection decision window; once a cystic structure is divided, even minimal subsequent dissection prompts the model to classify CVS as not achieved. Performance was consistently higher in low severity cases across all criteria, with significantly greater AUROC values in low versus high severity (criterion 1: 0.96 vs. 0.66, *p* < 0.001; criterion 2: 0.95 vs. 0.74, *p* < 0.001; criterion 3: 0.86 vs. 0.77, *p* = 0.03).

## Discussion

In this study, the AI platform demonstrated closer alignment with expert ground truth than narrative operative reports, as indicated by the higher discriminatory performance and greater reliability in classifying CVS attainment. This superiority of the platform was consistent across all disease-severity grades, underscoring its robustness in both straightforward and anatomically complex cases. These findings underscore the gap between perceived and video-based documentation: operative reports consistently overestimated CVS achievement, whereas the AI platform more accurately identified cases in which CVS was not attained. Despite its superior discriminatory performance, the platform’s reliability was moderate, which is expected given that expert multi-rater agreement was also moderate. The predominance of high-severity cases, clinical scenarios in which inflammatory changes and distorted anatomy increase subjectivity, further limits the attainable ceiling for chance-corrected agreement. Collectively, these findings support the use of video-based AI as an adjunct tool to complement surgeon documentation and to flag cases in which CVS was not achieved.

In contrast, narrative operative reports demonstrated poor reliability, likely reflecting several inherent biases [6, 11]. First, they are often completed retrospectively and represent surgeons’ perceptions, contributing to recall and subjectivity bias. Second, documentation is limited by template-based structures that assume full CVS achievement. Third, medico-legal considerations may unintentionally encourage overreporting. Accordingly, relying solely on narrative operative reports limits the accuracy of CVS documentation, while the addition of AI-based video analysis offers a more consistent documentation and reproducible framework [5, 19, 20].

Beyond documentation, the capabilities of AI-based analysis extend to a wide range of clinical and educational implications. In addition to reducing surgeons’ clerical workload and professional burnout, such AI platforms support continuous quality monitoring and targeted initiatives to improve CVS compliance [15]. Using AI-derived metrics may also provide feedback across experience levels, reinforce standardized techniques and help inculcate habits of safety from early training [21–24]. Furthermore, post hoc AI assessment may help identify clinical situations in which CVS was unlikely to be safely achieved, supporting educational review and reinforcing appropriate use of alternative strategies such as subtotal cholecystectomy or conversion. Alternative strategies and the rationale for aborting attempts at CVS achievement represent sound clinical judgment and not deviation from safety standards and should be clearly documented.

Looking ahead, AI models capable of real-time anatomical guidance and decision-support [25] may further enhance operative safety by identifying incomplete CVS exposure or imminent bile duct injury or, prompting immediate corrective actions and timely involvement of senior expertise or remote guidance [22]. As the literature on intraoperative AI continues to expand, several investigational models have emerged. Madani et al. developed [25] and Laplante et al. validated [26] a semantic segmentation model to identify a safe zone of dissection, although it does not specifically assess CVS. Mascagni et al. [27, 28] developed a platform that automatically extracts video clips to document timeframes preceding cystic duct division, but it does not evaluate CVS attainment. More recently, Murali et al. introduced a graph‑based deep learning approach capable of predicting CVS criteria from laparoscopic images by modeling anatomical relationships between structures. This work represents method-development combined with performance testing [29]. Kawamura et al. [30] described the development of an AI model for real‑time CVS assessment, reporting promising early results tested in a small cohort. Similarly, Petracchi et al. [31] prospectively tested, in real time, an AI model in 40 LCs, achieving complete concordance with expert evaluation of CVS; however, the cohort was limited to elective cases. A recent systematic review summarized the rapid progress of AI models developed for CVS and anatomic landmark identification during laparoscopic cholecystectomy. However, evidence remains constrained by heterogeneous methodologies, and small, selective cohorts [32].

In contrast, the present study evaluated a platform that is already implemented in routine clinical practice across several hospitals and countries. In this study, the platform was examined in the context of CVS documentation in a large real-world cohort, demonstrating its utility as an adjunct tool. A critical next step will be continued refinement to enhance accuracy, followed by robust multicenter validation and responsible integration into clinical workflows. In this context, recent global benchmarking efforts, most notably The Society of American Gastrointestinal and Endoscopic Surgeons (SAGES) CVS Challenge [33], established the first international benchmark for AI-assisted CVS assessment using 1,000 expert-annotated LC videos from thirteen participating teams. The challenge demonstrated meaningful methodological progress, including up to a 17% improvement in performance and over an 80% reduction in calibration error across participants. Within this setting, Theator achieved the strongest overall results, including accuracy, robustness, and uncertainty quantification. These findings highlight rapid advances in CVS-focused computer vision and underscore the growing maturity of AI systems designed to support operative safety.

Using AI platforms in surgery, however, carries potential legal and ethical implications if misinterpreted as replacing surgical judgment or serving as a stand-alone quality metric. Accordingly, such systems should be regarded strictly as decision-support and documentation-enhancement tools, with final responsibility fully remaining with the surgeon. Clear regulatory safeguards are needed to ensure appropriate use, such as stringent data-security for operative video, explicit definition of the platform’s intended use and clear delineation of professional and manufacturer liability, ongoing post-market performance monitoring and refinements, validation across diverse settings, and mandatory human-in-the-loop oversight. Nonetheless, intraoperative AI holds substantial promise for improving transparency, safety, and accuracy in surgery [34].

This study had several limitations. First, it was retrospectively designed and conducted at a single center, although the inclusion of many surgeons at different professional levels improved generalizability. Second, the study was conducted on the same group of quality initiative and therefore yielded higher rates of CVS achievement than reported in the literature. Nevertheless, the consistently low rate of accurate documentation when CVS was not achieved suggests minimal bias. Third, although the ground truth was based on consensus among three reviewers, one-quarter of cases required majority rather than unanimous agreement, indicating persistent subjectivity even when applying defined criteria. Expert multi-rater agreement was only moderate, and the predominance of high-severity cases, where interpretive variability is heightened, further constrained the attainable ceiling for chance-corrected agreement. Finally, because the reference standard is inherently subjective, the accuracy estimates reported here reflect concordance with expert consensus rather than absolute performance. Nevertheless, the AI model had been previously trained by the company on diverse external datasets and had no exposure to this cohort, supporting the interpretation that the observed accuracy reflects generalizability rather than cohort-specific optimization.

Despite these limitations, AI-based analysis offers a key advantage by applying consistent criteria across cases, while reducing human variability and the inherent bias in narrative documentation. Proper and fully transparent documentation of the CVS is essential for patient safety and from a clinical standpoint, could have implications on postoperative decision-making, particularly in cases where the CVS was not achieved. Such clinical implications may involve meticulous and longer in-hospital surveillance, and prompt investigation for early detection and treatment of postoperative complications. The platform’s high specificity enables accurate identification and flagging of these cases, unlike the low specificity of traditional documentation. Future studies should include consensus meetings to discuss interpretation of borderline cases to increase the reliability of expert ground truth, combined with large-scale prospective multicenter studies with a balanced representation of disease-severity sub-groups, to strengthen the robustness of the ground truth. Continuous refinement of the platform, including reconsideration of strict pre-dissection decision window, may reduce missed cases of adequate CVS, particularly when any dissection following a division of a cystic structure may lead the model to classify CVS as not achieved.

In conclusion, this study demonstrated an innovative and practical application of AI-based computer vision technology to address inaccuracies in operative documentation of the CVS. The AI-platform showed superior discriminatory performance compared with narrative documentation, consistent across disease-severity subgroups, supporting its benefit as an adjunct for CVS documentation. These findings provide support for adopting such platforms as adjuncts in routine practice, where implementation under appropriate human supervision may help create cross-border standardized reporting, enhance transparency, strengthen medico-legal accountability, and inform policy and training initiatives aimed at improving patient outcomes.

## Data Availability

Due to patient confidentiality and institutional restrictions on surgical video data, the datasets used in this study cannot be shared publicly. De-identified data may be made available from the corresponding author upon reasonable request and with appropriate institutional approvals.
